# Decanoic Acid Rescues Differences in AMPA-Mediated Calcium Rises in Hippocampal CA1 Astrocytes and Neurons in the 5xFAD Mouse Model of Alzheimer’s Disease

**DOI:** 10.3390/biom13101461

**Published:** 2023-09-27

**Authors:** Mina Abghari, Jenny Thythy Cecilia Mai Vu, Ninna Eckberg, Blanca I. Aldana, Kristi A. Kohlmeier

**Affiliations:** Drug Design and Pharmacology, Faculty of Health and Medical Sciences, University of Copenhagen, 2100 Copenhagen, Denmark; abgharimina@gmail.com (M.A.); jenny_vuu@hotmail.com (J.T.C.M.V.); ninnaeckberg@gmail.com (N.E.)

**Keywords:** Alzheimer’s disease, hippocampus, medium-chain fatty acids, diet, calcium

## Abstract

Alzheimer’s disease (AD), a devastating neurodegenerative disease characterized by cognitive dysfunctions, is associated with high levels of amyloid beta 42 (Aβ_42_), which is believed to play a role in cellular damage and signaling changes in AD. Decanoic acid has been shown to be therapeutic in AD. Glutamatergic signaling within neurons and astrocytes of the CA1 region of the hippocampus is critical in cognitive processes, and previous work has indicated deficiencies in this signaling in a mouse model of AD. In this study, we investigated glutamate-mediated signaling by evaluating AMPA-mediated calcium rises in female and male CA1 neurons and astrocytes in a mouse model of AD and examined the potential of decanoic acid to normalize this signaling. In brain slices from 5xFAD mice in which there are five mutations leading to increasing levels of Aβ_42_, AMPA-mediated calcium transients in CA1 neurons and astrocytes were significantly lower than that seen in wildtype controls in both females and males. Interestingly, incubation of 5xFAD slices in decanoic acid restored AMPA-mediated calcium levels in neurons and astrocytes in both females and males to levels indistinguishable from those seen in wildtype, whereas similar exposure to decanoic acid did not result in changes in AMPA-mediated transients in neurons or astrocytes in either sex in the wildtype. Our data indicate that one mechanism by which decanoic acid could improve cognitive functioning is through normalizing AMPA-mediated signaling in CA1 hippocampal cells.

## 1. Introduction

Alzheimer’s disease (AD) is responsible for 50–70% of all dementia cases [1]. AD is characterized by late-onset, progressive dementia associated with atrophy of the hippocampus and cerebral cortex, plaque formation of the protein beta-amyloid (Aβ), and intraneuronal neurofibrillary tangles of the protein tau [2]. AD typically begins with subtle memory failure that becomes more severe, and eventually, AD patients are incapacitated and unable to function without assistance. AD exhibits a sex-based risk, with females more likely than males to develop this disease [3,4,5].

While the mechanisms underlying AD are unknown, the AD brain is characterized by the presence of aggregations of Aβ, which is a proteolytic product of the amyloid precursor protein (APP). Elevated levels of Aβ have been suggested to be caused by genetic mutations in the genes for amyloid precursor protein (APP), presenilin 1 (PS1), and presenilin 2 (PS2) as early onset and familial forms of AD have been associated with mutations in these genes [6,7,8]. Amyloid plaques, which are aggregations of the Aβ peptide, are the basis of the amyloid hypothesis of AD [9,10]. The Aβ hypothesis suggests that the pathological amyloidogenic effect is related to the length of amino acids in the proteolytic product. In cases of autosomal genetic mutations in the genes for APP, PS1, and PS2, the production of Aβ is altered, and the Aβ peptide with a length of 42 amino acids (Aβ_42_) is the major product, suggesting that Aβ_42_ is responsible for amyloidosis [7,8]. Formation of Aβ_42_ into extracellular amyloid plaques likely induces the release of free radicals and alters the activity of kinases and phosphatases, causing neuronal dysfunction and neuronal death.

Biomarkers of AD are amyloidosis and neurodegeneration, the latter being characterized by hippocampal atrophy [7,8,10,11]. However, following results using PET scanning that regional glucose use was reduced in senile dementia [12], recognition has emerged that disturbances in the cerebral metabolic rate of glucose (CMRglu) are present in early-stage AD, in mild cognitive impairment and in adults with genetic factors enhancing their risk of AD [13,14,15,16,17]. In a healthy brain, the primary energy source is glucose; however, in AD, downregulation of the GLUT-1 and GLUT-3 transporter, which transport glucose across the BBB, occurs, resulting in a state of hypometabolism [18]. Alterations in CMRglu are now seen as an additional reliable biomarker for the progression of AD [14,15,16].

Changes in glucose metabolism appear very early in the chain of pathological causative events, and interestingly, early provision of energy to the brain limits the deposition of Aβ [19]. During a glucose hypometabolic state, the brain is able to utilize alternative sources of energy to sustain ATP production. Two such sources are amino acids and ketone bodies [20], and while glucose uptake is impaired in AD patients, utilization of amino acids and ketones is unaffected [13]. Major metabolites resulting from the ketogenic diet, which has been popularized as a treatment for AD, include ketone bodies, and this diet has proved to provide some cognitive benefits [21,22,23,24]. However, increasingly, the focus has shifted to supplementation with medium-chain fatty acids (MCFA), which, in addition to their metabolic effect following conversion into ketone bodies by processes in the liver [25], could exert independent functional actions through interactions with cellular proteins. MCFAs, which are saturated fatty acids with a chain length varying from 6–12 carbons and a carboxylic head group, have been shown to be therapeutic in models of AD [21], and we and others have shown a direct effect of MCFAs on astrocyte energy metabolism [13,26,27].

Decanoic acid is a MCFA with a chain length of 10 carbon atoms, which has been shown to stimulate glycolysis, producing lactate available as fuel for neural cells [26]. We have shown decanoic acid can affect astrocyte energy metabolism, which could also improve neural function [27]. In addition, decanoic acid has been shown to exhibit anti-inflammatory properties [28]. Excitingly, supplementation with decanoic acid improved mild cognitive impairment (MCI) and other cognitive symptoms associated with the early stages of AD, which has led to the suggestion that decanoic acid could delay the onset and progression of AD [29]. However, while there is evidence, albeit limited, that decanoic acid could be beneficial for cognitive functioning, few cellular studies have been conducted in mouse models of AD to explore the functional effects of decanoic acid. Accordingly, in this study, we examined the effects of decanoic acid on hippocampal cellular activity in a mouse model of AD. We used the 5xFAD transgenic mouse in which the level of Aβ_42_ increases in a linear manner as 5xFAD mice age, which is associated with neural damage and non-specific reactive changes in glia cells (gliosis) [8]. Further, sex-based differences in the 5xFAD mouse model have been observed in that female 5xFAD mice exhibit an earlier onset of Aβ pathology and a more robust induction of inflammatory mediators compared to male 5xFAD mice [30]. Therefore, we evaluated whether sex differences existed in the effects of the 5xFAD mutations on CA1 hippocampal function and whether decanoic acid exerted differential actions.

## 2. Methods

### 2.1. Animals

All animal experiments were approved by the Animal Welfare Committee appointed by the Danish Ministry of Justice and in accordance with the European Communities Council Directive of 22 September 2010 (2010/63/EU) on the Protection of Animals Used for Experimental and Other Scientific Purposes. Handling of the animals was performed by authorized personnel at the Department of Drug Design and Pharmacology at the University of Copenhagen. This study included use of the 5xFAD transgenic mouse of AD, which has five mutations associated with familial AD. For this study, transgenic female and male 5xFAD mouse APP_K670N/M671L,I716V,V717I_, PS1_M146L,L286V_, Jax strain: 006554) and female and male wildtype (WT) mice (Jax strain: 100012) on the same B6/SJLF1J background were used which were purchased from Jackson Laboratories (Bar Harbor, ME, USA). A colony of these mice was bred and maintained at the Department of Drug Design and Pharmacology at the University of Copenhagen. Mice were genotyped from tale clippings by standard PCR protocol (Jax protocol: 23370) for the APP gene using the primers: Transgene forward: AGG ACT GAC CAC TCG ACCAG (o1MR3610), transgene reverse: CGG GGG TCT AGT TCT GCA T (o1MR7338), internal positive control reverse: GTA GGT GGA AAT TCT AGC ATC ATC C (o1MR7339). For our first study, one cohort of male and female animals was used at 10–22 postnatal days of age, and data were combined. For subsequent studies that evaluated sex as a factor, male and female mice of 8–10 weeks of age were used. Animals were housed in a humidity and temperature-controlled room (temperature 22 °C and humidity 36–58%) with a 12:12 h light/dark cycle starting from 7 a.m. to 7 p.m. with ad libitum access to water and food.

### 2.2. Brain Slices

Brains were extracted from mice that had been deeply anesthetized with isoflurane (Attane Vet, ScanVet Animal Health A/S, Fredensborg, Denmark) as evaluated by loss of reflexes to a paw pinch. Following decapitation, the brain was submerged, and brain slices were cut in two different versions of artificial cerebrospinal fluid (aCSF), depending on age of the animal. Submersion of the brain and preparation of younger brain slices was conducted in aCSF composed of NaCl (124 mM), KCl (5 mM), NaHCO_3_ (26 mM), Na_2_HPO_4_ (1.2 mM), glucose (10 mM), CaCl_2_ (2.7 mM) and MgSO_4_ (1.2 mM). Prior to adjustment of pH and osmolarity to 7.40 (±0.05) and 300 (±5) mOsm, the solution was bubbled with carbogen (95% O_2_/5% CO_2_) for 30 min. Preparation of slices from animals older than 22 days was performed in aCSF containing KCl (2.5 mM), NaHCO_3_ (30mM), Na_2_HPO_4_ (1.2 mM), glucose (25 mM), CaCl_2_ (0.5 mM), MgSO_4_ (1.2 mM), 4-(2-hydroxyethyl)-1-piperazineethanesulfonic acid (HEPES) (20 mM), sodium ascorbate (5 mM), n-methyl D-glucamine (NMDG) (93 mM), Sodium pyruvate (3 mM) and Thiourea (2 mM). This NMDG-based solution was bubbled with carbogen (95% O_2_/5% CO_2_) for 30 min, followed by adjustment of pH and osmolarity to 7.30 (±0.05) and 300 (±5) mOsm, respectively. A block of the brain containing the hippocampus was mounted on the stage of a vibracheck-calibrated Leica vibratome (VT1200S, Leica Biosystems, Nußloch, Germany), and coronal brain slices containing the CA1 region of the hippocampus were sectioned at a thickness of 250 μm. The cutting speed was set to 0.06–0.09 mm/s depending on the age of the mouse, with younger mice requiring a lower blade speed, and the horizontal amplitude was set to 1 mm. The sectioning process was performed in a precooled chamber containing a slushy mixture of ice and liquid aCSF, which were oxygenated throughout the slicing. Slices of 250 µM were incubated in a preheated chamber in a water bath set at 36 °C for 15 min and then placed at room temperature (20–22 °C) for acclimatization for 1 h under constant supply of carbogen before continuing with further procedures.

### 2.3. Hippocampal CA1 Cell Loading with SR101 and Fura-2 AM

Hippocampal brain slices were exposed to sulforhodamine 101 (SR101), which is a fluorescent dye that can be used to visualize astrocytes in the CA1 region of the hippocampus [31]. A tube containing 2.5 μL of 5 mM SR101 (in dimethylsulfoxid (DMSO)) was added to 500 μL of aCSF, light protected, and vortexed. One-half of the hippocampal brain slices (containing one side of the hippocampus) were transferred to this tube, and the slice was incubated in a water bath at a temperature of 32 °C for 20 min. Following SR101 loading, the slice was placed in the slice holding chamber and washed with aCSF for 10 min. Following this, 2.5 μL of 4 mM Fura-2 AM (in DMSO) was combined with 2.5 μL Pluronic acid ^®^ F-127 (10% *w*/*v* solution in water, Life Technologies, Carlsbad, CA, USA) and 500 μL aCSF and vortexed for 2 min. The solution was transferred to a small test tube, which was light-protected, and the SR101-loaded slice was transferred from the slice-holding chamber to this tube. The tube was incubated in a heated water bath at a temperature of 32 °C. The incubation time for the slices was calculated as the age of the animal in days converted to minutes plus 10 min [32]. Neurons selected for recordings were identified as large cells (>17 µM) positive for Fura-2 but negative for SR101, whereas astrocytes selected for recordings were identified as positive for both Fura-2 and SR101.

### 2.4. Decanoic Acid Pre-Incubation and Acute AMPA Exposures

Slices were incubated for one hour in 100 mL of 200 µM decanoic acid (NEOBEE 1095, Stephan, Northfield, IL, USA) under constant carbogen exposure and light protection. AMPA was applied to the slices through the bath aCSF with a perfusion rate of 1.2 mL/min delivered via a perfusion pump (Ole Dich, Hvidovre, Denmark). Stock solutions of 10 mM AMPA (Tocris, Bristol, UK) stored in aliquots at −20 °C were prepared right before use by adding to aCSF. The final concentration and volume of AMPA, which was applied via the bath, was 10 μM and 3 mL, respectively.

### 2.5. Calcium Imaging

Fluorescent-based imaging was performed on the hippocampal slices to investigate changes in fluorescence of Fura-2 in SR-101 positive (putative astrocytes) and SR-101 negative cells (putative neurons) upon drug application. Dye-loaded slices were moved to a recording chamber, which was attached to an Olympus BX-50 microscope. The CA1 region of the hippocampus was identified under bright field optics under a 4× objective lens (Plan N, Olympus, Tokyo, Japan). The CA1 was identified based on anatomical landmarks, which are illustrated in Paxinos and Franklin’s mouse brain atlas [33]; specifically, the landmarks were the dentate gyrus, the alveus, and the external capsule. Once located, a 40× water immersion lens was used to identify cells (LUMPlanF N, Olympus, Japan). Following the identification of cells in the CA1, illuminated light was extinguished, and fluorescent optics using a Xenon bulb operated by a monochromator (Polychrome V, FEI Munich GmbH, Gräfelfing, Germany) were applied. Appropriate filter sets for Fura-2 AM and SR101 were used (Chroma Fura-2 filter and 594 nM) with excitation at appropriate wavelengths under software control (Live Acquisition, Till Vision, FEI, Munich GmbH, Gräfelfing, Germany). To visualise SR101-loaded cells, slices were excited at a wavelength of 570 nm. To visualise Fura-2-loaded cells, slices were excited at 340 nm and 380 nm, with emission measured at 515 nm. Optical recordings were captured by a 12 bit CCD camera system (Sensicam 382KL1090, PCO AG, Kelheim, Lower Bavaria, Germany) attached to the microscope and controlled by software (Live Acquisition, Till Photonics, Victor, NY, USA). A region of interest (ROI) was drawn around cells. Fluorescence within an area devoid of cells was used as background fluorescence. Images were binned at 2 × 2, which balanced the degree of temporal and spatial resolution, and image pairs (340/380 nm) were captured at a frequency of 2 s with an exposure time of 75–150 milliseconds.

Raw data obtained from the LA software were evaluated by using the Offline Analysis (OA) software (Till Vision, Germany). If cells moved out of the marked ROI due to tissue movement, they were eliminated from analysis. Cells were also excluded if excessive Fura-2 particles flowed in the ROIs or if excessive rapid changes in fluorescence were noted at baseline, which would obscure drug effects. The average fluorescence within each ROI was obtained for each image pair, background fluorescence was subtracted, and a ratio of fluorescence was calculated. The average baseline fluorescence was determined from an average of 10 image pairs collected before AMPA exposure to the slice and subtracted from the entire recording, which allowed the data to be converted to changes relative to baseline. Data are plotted as a function of time (in seconds) with “DF” denoting the fluorescence ratio of a time point in which baseline fluorescence has been subtracted, and the entire run has been normalized to baseline fluorescence, allowing conversion to a percentage (%DF/F). In figures, upward deflections indicate rises in intracellular calcium levels. The peak effect of AMPA is calculated from the average deflection from baseline of 10 data points during drug actions. The amplitude of responses is defined as the difference between the average baseline (DF/F) value and the average peak value, and amplitudes were only calculated from responding cells with very stable baselines, clearly defined response peaks, and minimal photobleaching. Data obtained from OA were transferred to Microsoft Excel (Microsoft Office) to calculate the amplitudes of responses in percentage (%DF/F).

### 2.6. Statistical Analysis and Data Presentation

All statistical analyses were conducted using GraphPad Prism Version 9.2 (GraphPad Software, San Diego, CA, USA). The ROUT method was used to identify significant outliers in the calculated amplitudes for each dataset. The outliers were excluded according to the aggressivity constant (Q-value) of 1%. Furthermore, the D’Agostino and Pearson test was used to test the data for normal distribution. Data sets were considered normally distributed if the probability value (*p*) was *p* > 0.05. The appropriate test for comparison was then applied. When comparing two different groups of cells and if the data passed the normality distribution test, then an unpaired Student’s *t*-test was used. If the normality test was not passed, a Mann–Whitney test was used. When more than two groups were compared, a one-way ANOVA test was used, but a two-way ANOVA was used to determine whether there was an interaction between sex and treatment in the 5xFAD animals. If the ANOVA result indicated a significant difference, the test was followed by Tukey’s Honestly Significant Difference (HSD) post hoc test to determine between which columns the differences were present. Furthermore, a Fisher’s exact test (two-sided) was applied for comparison of categorical data, i.e., the proportion of responding and non-responding cells. For all statistical analyses performed, the statistical significance was indicated by the p-value and considered significant if *p* < 0.05. The level of significance is indicated by asterisks denoted as: *: *p* < 0.05, **: *p* < 0.01, ***: *p* < 0.001, ****: *p* < 0.0001. All graphs presented in the result section were created in GraphPad Prism 9.2. Changes in fluorescence, suggestive of changes in intracellular calcium, are presented as %DF/F over time in seconds. All the figures show upwards deflection in %DF/F indicative of a relative increase in intracellular calcium levels. No downward deflections in %DF/F were observed. For all groups of data, the number of cells is denoted as n and is derived from slices taken from mice from at least 3 different litters. All data are presented as mean ± standard error of the mean (SEM).

## 3. Results

### 3.1. AMPA-Induced Changes in Fluorescence in Hippocampal CA1 Neurons and Astrocytes

**Figure 1:**
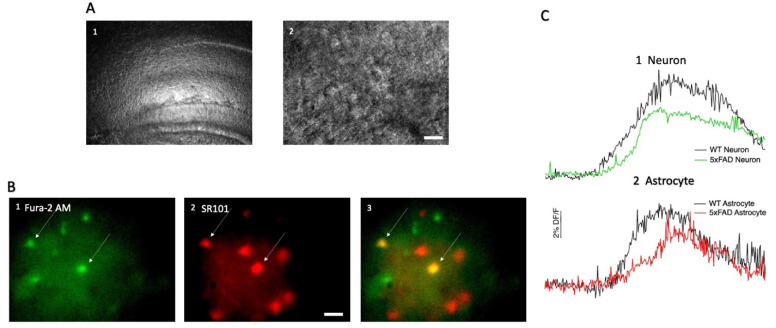
Recordings were performed from astrocytes and neurons in the CA1 region of the hippocampus (**A1**) Under bright field optics, the structure of the CA1 region of the hippocampus can be seen under 4× magnification in mouse brain slices. (**A2**) Under 40× magnification and bright field optics, individual cells are apparent. (**B1**) Representative fluorescent image of cells loaded with the intracellular calcium indicator Fura-2 AM under 380 nm wavelength light is shown. (**B2**) The same field is shown under 560 nm fluorescent light in which astrocytes loaded with the astrocytic marker sulforhodamine 101 (SR101) can be seen. (**B3**) In the merged image, cells positive for SR101 and Fura-2 are marked with arrows and determined to be astrocytes. Scalebar in A and B indicates 20 µm. (**C**) AMPA induces rises in fluorescence in Fura-2 AM loaded cells, which are indirect indicators of increases in calcium as shown in these representative responses from hippocampal CA1 neurons (**C1**) and CA1 astrocytes (**C2**) in the hippocampus of the 5xFAD (green and red traces in **C1**,**C2**) and WT hippocampus (black traces in **C1**,**C2**) when AMPA was applied. The amplitude of the change in %DF/F from baseline induced by AMPA was compared across genotypes in the two cell types.

We wished to compare rises in calcium induced by AMPA in the neurons and astrocytes of the CA1 region of the hippocampus between the 5xFAD and control animals. In order to distinguish the two different cell types, astrocytes were identified by co-loading hippocampal brain slices with Fura 2-AM and SR101, which is selectively taken up by astrocytes (Figure 1A,B). Unsurprisingly, AMPA induced a change in fluorescence in the majority of both neurons and astrocytes in the CA1 region of both 5xFAD and WT controls, indicative of rises in calcium (Figure 1(C1,C2)). Upon gross examination by an investigator who was not blinded to genotype, in our first cohort of animals, which was comprised of nine 5xFAD animals and eleven WT mixed-sex animals reared side by side with the 5xFAD animals, there were no kinetic differences in responses elicited in these two different cell types (Figure 1); however, this point was not a focus of the study, so a detailed comparison was not conducted, and our focus was on examination of numbers of cells responding and the amplitude of the rise in fluorescence.

### 3.2. The Proportion of Responding Neurons and Astrocytes in Hippocampal CA1 Did Not Differ between 5xFAD and WT

**Figure 2:**
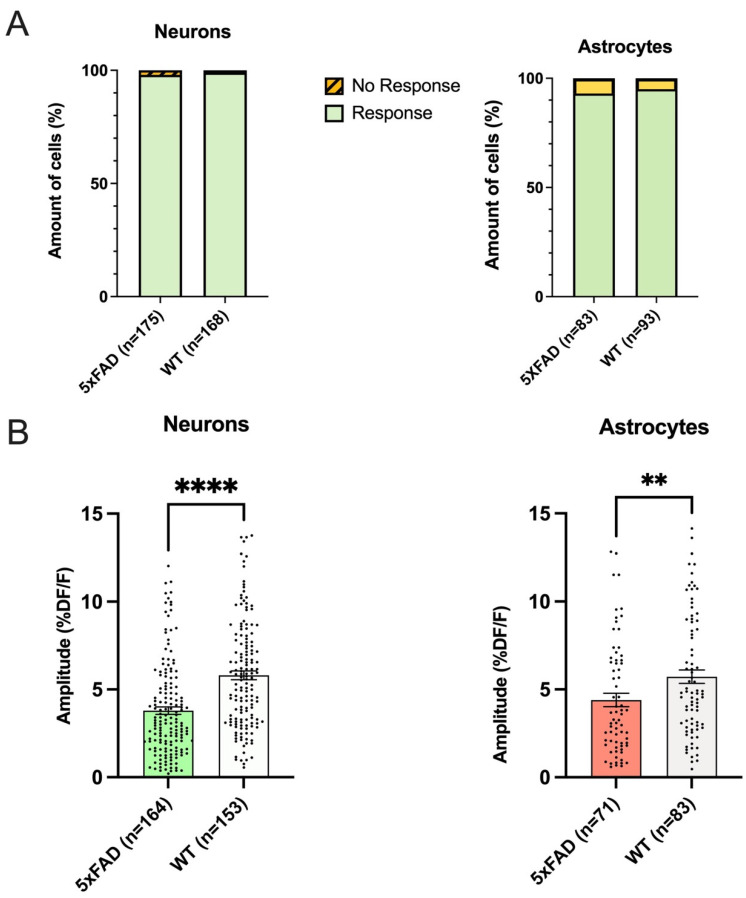
Smaller rises in calcium are elicited in both hippocampal neurons and astrocytes in one-month-old 5xFAD mice compared to rises seen in wildtype (WT) (**A**) There were no differences in the proportions of neurons or astrocytes responding to AMPA with rises in fluorescence in the CA1 hippocampal region between male and female one-month-old 5xFAD and WT mice (10-22 PND). (**B**) One-month-old 5xFAD mice exhibited significantly smaller fluorescent changes in response to AMPA than WT in both hippocampal neurons and astrocytes, as shown in the population data. Histograms compare the AMPA-induced fluorescence changes to 10 μM AMPA between the population of 5xFAD and WT neurons (Mann–Whitney test: *p* < 0.0001) and astrocytes (Mann–Whitney test: *p* = 0.0097), indicating smaller rises in calcium in both cell types in the 5xFAD. Amplitude of the response is presented as %DF/F in this and subsequent figures. In this and subsequent figures, the number of cells is indicated by n in the x-axis, and the significance level is presented above the bars with asterisks. In this figure, ** *p* < 0.01 and **** *p* < 0.0001.

As this is the first study to compare the effects on intracellular calcium levels by bath application of AMPA in cells in the dorsal CA1 region of the hippocampus in the 5xFAD mouse, our initial examination in a one-month-old cohort of animals of mixed sex was focused on whether there was a difference in the proportion of cells responding to AMPA with rises in fluorescence in this area of the brain. There was not a genotype difference in responsive and non-responsive neurons in hippocampal CA1 as 98% and 99% of 5xFAD and WT neurons tested responded, respectively, which did not represent a statistical difference (5xFAD: Responded n = 171, No response n = 4; WT: Responded n = 167, No response n = 1; Fisher’s Exact Text *p* = 0.3719; Figure 2A). There was no difference in the proportion of responsive and unresponsive astrocytes between 5xFAD and WT mice as 93% and 95% of examined cells responded in both genotypes (5xFAD: Responded n = 77, No response n = 6; WT: Responded n = 88, No response n = 5; Fisher’s Exact Text *p* = 0.7579; Figure 2A).

### 3.3. Both Astrocytes and Neurons in 5xFAD Mice Exhibited Smaller AMPA-Induced Calcium Responses Than WT

As the proportions of cells that responded to AMPA with rises in calcium did not differ, our next analysis was to examine whether the amplitude of the rises differed in 5xFAD CA1 cells when compared to those seen in WT cells, as this could suggest a change in AMPA receptor-mediated functionality. Interestingly, the average amplitude of the calcium rise induced by AMPA in the 5xFAD CA1 neurons was 34% smaller than that seen in the WT. In the 5xFAD group, the average amplitude of the change in fluorescence was 3.8 ± 0.2 %DF/F (*n* = 164), and in the WT group, the average increase was 5.8 ± 0.2 %DF/F (n = 153), which represented a significant difference (Mann–Whitney test: *p* < 0.0001; Figure 2B). When the amplitudes were compared across genotypes in astrocytes, in results which were qualitatively similar to what was seen in neurons, there was a 17% smaller response in AMPA-mediated calcium responses in 5xFAD animals compared to WT. In the 5xFAD group, the average amplitude of the increase in calcium in astrocytes was 4.4 ± 0.4 %DF/F (n = 71 cells), and in the WT group, the average amplitude was 5.3 ± 0.4 %DF/F (n = 83 cells) which was significantly different (Mann–Whitney test: *p* = 0.0097; Figure 2B).

### 3.4. Smaller AMPA-Mediated Calcium Rises in 5XFAD Neurons and Astrocytes Were Seen in Both Sexes

We had previously noted changes in hippocampal excitatory neuronal activity, which could have led to larger glutamate-mediated calcium rises in the 5xFAD male hippocampus at two months of age [34]. Accordingly, we wished to examine whether smaller AMPA-mediated calcium transients were also seen in two-month-old 5xFAD when compared to age-matched WTs. Further, we wished to determine whether any sex-based differences existed in AMPA transients in neurons and astrocytes within genotypes. Accordingly, in a new cohort of 5xFAD and WT animals, which were between 8–10 weeks of age and were housed under identical stable conditions, we repeated our study to evaluate the proportion of responding cells and the amplitude of responses, and we conducted a separate analysis of male and female data. For these studies, experiments were interleaved between 5xFAD and WT animals with unblinding of the investigator to genotype only after analysis was completed. In this study, a total of twenty-four female mice (5xFAD:15, WT:9) and twenty-three male mice were used (5xFAD:10, WT:13).

**Figure 3:**
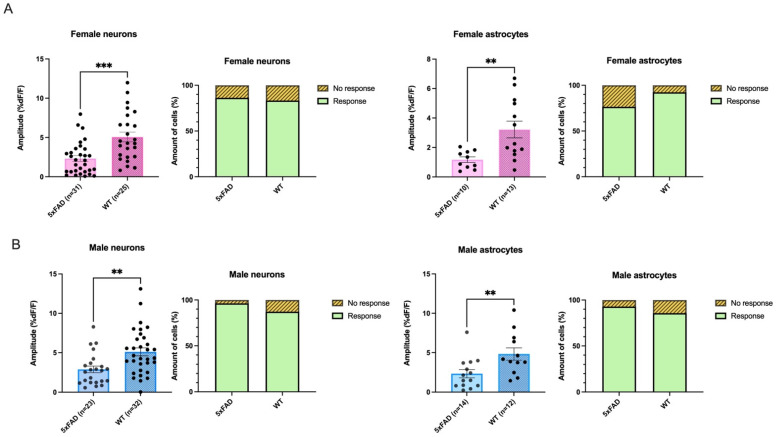
Smaller AMPA-induced rises were elicited in both females and males in neurons and astrocytes in the 5xFAD genotype. There were no differences in either females (**A**) or males (**B**) in the proportions of hippocampal neurons or astrocytes responding to AMPA with rises in fluorescence between two-month-old 5xFAD and wildtype (WT) mice. (**A**,**B**) Two-month-old female and male 5xFAD mice exhibited significantly smaller amplitude fluorescent changes in response to 10 μM AMPA than WT in both hippocampal neurons (Female: Mann–Whitney test: *p* = 0.0002; Male: Mann–Whitney test: *p* = 0.0015) and astrocytes (Female: Mann–Whitney test: *p* = 0.0053; Male: Mann–Whitney test: *p* = 0.0077) as shown in the population data. The significance level is presented above the bars with asterisks denoting ** *p* < 0.01 and *** *p* < 0.001.

Reflecting what had been seen in earlier recordings in the one-month-old cohort, there were no differences in the proportion of neurons responding between genotypes in either females or males in the 8- to 10-week-old group (Female 5xFAD vs. Female WT: 5xFAD: Response n = 44, No response n = 7. WT: Response n = 25, No response = 5, Fisher’s exact test: *p* = 0.7533; Male 5xFAD vs. Male WT; 5xFAD: Response n = 26, No response n = 1. WT: Response n = 34, No response = 5, Fisher’s exact test: *p* = 0.3880; Figure 3A,B). Neither was there a significant genotype difference between responding astrocytes in females or in males (Female 5xFAD vs. Female WT: 5xFAD: Response n = 13, No response n = 4, WT: Response n = 13, No response = 1, Fisher’s exact test: *p* = 0.3445; Male 5xFAD vs. Male WT: 5xFAD: Response n = 14, No response n = 1. WT: Response n = 12, No response n = 2, Fisher’s exact test: *p* = 0.5977; Figure 3A,B).

We next examined the amplitude of AMPA-mediated calcium transients. Similar to what had been seen in one-month-old animals, in two-month-old animals, the rises seen in both astrocytes and neurons of the 5xFAD animals were smaller than those seen in WT. Further, as we had conducted a separate sex study, we were able to note that smaller rises were elicited in both females and males. Specifically, in female 5xFAD mice, a 54.2% lower AMPA-mediated Ca^2+^ response was elicited in 5xFAD neurons compared to that seen in the WT, which was significant (5xFAD: 2.32 ± 0.4 %DF/F, n = 31 neurons, WT: 5.07 ± 0.6 %DF/F, n = 25; Mann–Whitney test: *p* = 0.0002; Figure 3A). In astrocytes, a similar pattern was seen with transients in female 5xFAD being 64% smaller than those elicited in female WT, which was a significant difference (Average amplitude: 5xFAD: 1.17% ± 0.2 %DF/F, n = 10, WT: 3.22 ± 0.6 %DF/F, n = 13, Mann-Whitney test: *p* = 0.0053; Figure 3A).

Similarly, a significant difference was shown in neurons between male 5xFAD mice and male WT mice, as responses were 43% lower in 5xFAD mice (average amplitudes: 5xFAD: 2.90 ± 0.4 %DF/F, n = 23, WT: 5.10 ± 0.5 %DF/F, n = 32, Mann–Whitney test: *p* = 0.0015; Figure 3B). A significant difference was also seen in astrocytes between 5xFAD and WT male mice with 52% lower responses elicited in 5xFAD (average amplitudes: 5xFAD: 2.34 ± 0.5 %DF/F, n = 14, WT: 4.83 ± 0.8 %DF/F, n = 12, Mann–Whitney test: *p* = 0.0077; Figure 3B).

### 3.5. AMPA-Mediated Calcium Transients Do Not Exhibit a Sex-Based Difference in 5xFAD or WT CA1 Neurons

**Figure 4:**
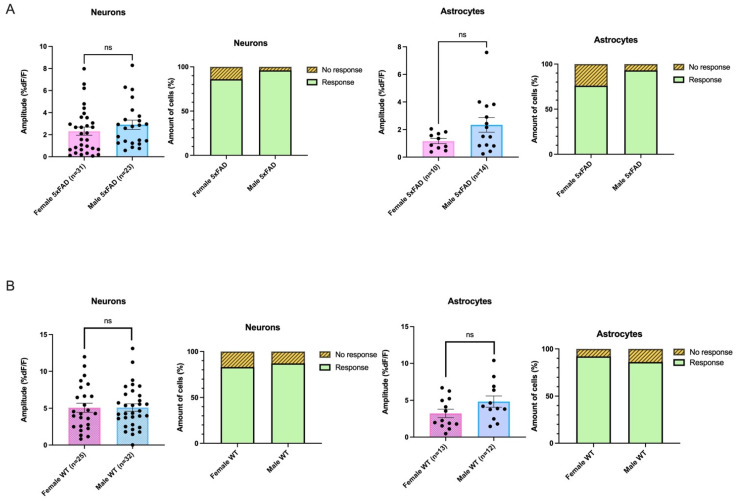
The proportion of neurons and astrocytes that responded to AMPA and the amplitude of the responses did not differ between females or males irrespective of genotype. (**A**) The amplitude of the AMPA-induced calcium rises in neurons and astrocytes in the 5xFAD genotype and the proportion of each cell type that responded to AMPA with rises in calcium did not differ between females and males. (**B**) Similarly, the proportion of neurons and astrocytes that responded to AMPA in the WT and the amplitude of the change in fluorescence did not differ between females and males. ns denotes no significance.

In the 5xFAD animals, there was no sex-based difference in the amplitude of AMPA-mediated transients in neurons, as the amplitude elicited by AMPA in females did not differ from that elicited in males (average amplitudes: Female 5xFAD: 2.32 ± 0.4 %DF/F, n = 31, Male 5xFAD: 2.90 ± 0.4 %DF/F, n = 23, Mann–Whitney test: *p* = 0.1838; Figure 4A). The proportion of neurons responding in 5xFAD females also did not show a significant difference from that responding in 5xFAD males (Female 5xFAD: response n = 44, No response n = 7, Male 5xFAD: response n = 26, No response n = 1, Fisher’s Exact test, *p* = 0.2503; Figure 4A).

Further, there was no sex-based difference in the average amplitude of the AMPA-mediated change in fluorescence induced in the astrocytes of the 5xFAD female compared to that seen in the 5xFAD male (average amplitudes: Female 5xFAD: 1.17 ± 0.2 %DF/F, n = 10, Male 5xFAD: 2.34 ± 0.5 %DF/F, n = 14; Mann–Whitney test: *p* = 0.1375; Figure 4A). The proportion of responses seen in astrocytes in female 5xFAD animals was not different than that seen in 5xFAD males (Female 5xFAD: response n = 13, No response n = 4, Male 5xFAD: response n = 14, No response n = 1, Fisher’s Exact test, *p* = 0.3382; Figure 4A).

AMPA-mediated transients in neurons and astrocytes in WT females also did not significantly differ from that elicited in WT males as far as amplitude (neurons: average amplitude: Female WT control: 5.07 ± 0.6 %DF/F, n = 25. Male WT control: 5.10 ± 0.5 %DF/F, n = 32, Unpaired *t*-test: *p* = 0.9631; astrocytes: average amplitude: Female WT control: 3.22 ± 0.6 %DF/F, n = 13. Male WT control: 4.83 ± 0.8 %DF/F, n = 12, Unpaired *t*-test: *p* = 0.1038; Figure 4B). The proportion responding in the WTs also did not differ between females and males (neurons: Female WT control: Response n = 25, No response n = 5, Male WT control: Response n = 34, No response n = 5, Fisher’s Exact Test, *p* = 0.7370; astrocytes: Female WT control: Response n = 13, No response n = 1, Male WT control: Response n = 12, No response n = 2, Fisher’s Exact Test, *p* = 1.000; Figure 4B). When taken together, our data suggest that significant differences in AMPA-mediated calcium responses in neurons and astrocytes seen in our first cohort of mixed-sex one-month-old 5xFAD and WT animals were not driven by one sex.

### 3.6. Decanoic Acid Pre-Treatment Results in Significantly Heightened Calcium Responses in 5xFAD Astrocytes and Neurons in Male and Females

In our next series of experiments, we wished to examine whether semi-acute treatment with decanoic acid would affect neuronal and astrocyte calcium responses to AMPA in the CA1. While we showed that AMPA induced similar amplitudes of AMPA-mediated calcium rises in the 5xFAD male and female, and that rises were also not different in the WT, it was possible decanoic acid-induced sex-based differential effects; therefore, we continued to conduct our study with females and males analyzed as separate groups. We compared the effect of preincubation of slices in decanoic acid for one hour prior to the application of AMPA to see if induced calcium rises were different than in the absence of this MCFA.

**Figure 5:**
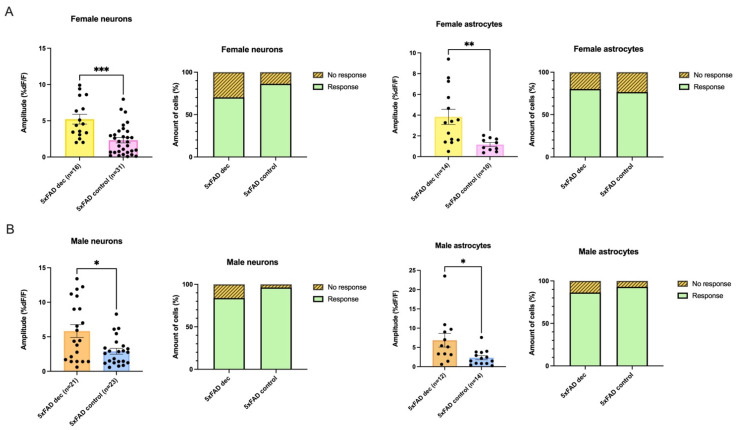
Decanoic acid resulted in significantly larger AMPA-mediated transients in the 5xFAD genotype in neurons and astrocytes in both females and males. (**A**) While the proportion of neurons and astrocytes responding to AMPA in slices that had been incubated in decanoic acid did not differ from control conditions, the amplitude of AMPA-mediated calcium rises was significantly greater in both cell types in slices exposed to decanoic acid from the female (neurons: Mann–Whitney test: *p* = 0.0003; astrocytes: Mann–Whitney test: *p* = 0.0038). (**B**) The amplitude of AMPA-mediated rises in calcium was also greater in neurons and astrocytes from the 5xFAD male that had been preincubated in decanoic acid (neurons: Mann–Whitney test: *p* = 0.0216; astrocytes: Mann–Whitney test: *p* = 0.0127). The significance level is presented above the bars with asterisks denoting * *p* < 0.05, ** *p* < 0.01, and *** *p* < 0.001.

The proportion of neurons responding in the 5xFAD female and male did not significantly differ when conditions of the presence of decanoic acid were compared to those in its absence (neurons: Female 5xFAD_Dec_ vs. Female 5xFAD_Control_, Female 5xFAD_Dec_: Response n = 26, No response n = 11. Female 5xFAD_Control_: Response n = 44, No response n = 7, Fisher’s exact test: *p* = 0.1067; neurons: Male 5xFAD_Dec_ vs. Male 5xFAD_Control_, 5xFAD_Dec_: Response n = 26, No response n = 5. 5xFAD_Control_: Response n = 26, No response n = 1, Fisher’s exact test: *p* = 0.2007; Figure 5A,B). Similarly, there were no differences in the proportion of astrocytes responding in the 5xFAD female or male when decanoic acid was present when compared to proportions in its absence (astrocytes: Female 5xFAD_Dec_ vs. Female 5xFAD_Control_, 5xFAD_Dec_: Response n = 16, No response n = 4. 5xFAD_Control_: Response n = 13, No response n = 4, Fisher’s exact test: *p* = 1.000; Male 5xFAD_Dec_ vs. Male 5xFAD_Control_, 5xFAD_Dec_: Response n = 19, No response n = 3. 5xFAD_Control_: Response n = 14, No response = 1, Fisher’s exact test: *p* = 0.6328; Figure 5A,B).

While there were no differences in the proportion of cells responding in 5xFAD females or males dependent on pretreatment with decanoic acid, remarkably, the amplitude of response to AMPA was significantly higher in the group of neurons in which slices had been incubated in decanoic acid compared to the amplitude seen in the non-incubated slices from the 5xFAD mouse. This effect of decanoic acid was present in both females and males (Figure 5A,B).

In females, the average amplitude of the rise seen in neurons of the 5xFAD animal exposed to decanoic acid was more than double that seen in non-incubated conditions, which was a significant difference (5xFAD_Dec_: 5.21 ± 0.7 %DF/F, n = 16, 5xFAD_Control_: 2.32 ± 0.4 %DF/F, n = 31, Mann–Whitney test: *p* = 0.0003; Figure 5A). An effect of decanoic acid was also seen in astrocytes as the amplitude of the AMPA-induced rise was 3-fold higher in female 5xFAD astrocytes from slices that had been preincubated in decanoic acid when compared to 5xFAD astrocytes which were not pre-exposed. This difference was significant (average amplitudes: 5xFAD_Dec_: 3.83 ± 0.7 %DF/F, n = 14, 5xFAD_Control_: 1.17 ± 0.2 %DF/F, n = 10, Mann–Whitney test: *p* = 0.0038; Figure 5A).

In males, a similar effect on neurons was seen in slices exposed to decanoic acid. Preincubation in decanoic acid was associated with a response to AMPA in 5xFAD neurons that was double that seen in non-incubated conditions, which represented a significant difference (Average amplitudes: 5xFAD_Dec_: 5.84 ± 0.9 %DF/F, n = 21, 5xFAD_Control_: 2.90 ± 0.4 %DF/F, n = 23, Mann–Whitney test: *p* = 0.0216; Figure 5B). Preincubation of slices in decanoic acid was also associated with a nearly 3-fold higher AMPA-mediated change in fluorescence in astrocytes, which is a significant difference from the amplitude seen in 5xFAD astrocytes without preincubation (average amplitudes: 5xFAD_Dec_: 6.87 ± 1.8 %DF/F, n = 12, 5xFAD_Control_: 2.34 ± 0.5 %DF/F, n = 14, Mann–Whitney test: *p* = 0.0127; Figure 5B).

### 3.7. Decanoic Acid Pre-Treatment Does Not Result in a Significantly Different Average Amplitude of AMPA-Mediated Transients between Males and Females in the 5xFAD Hippocampus

**Figure 6:**
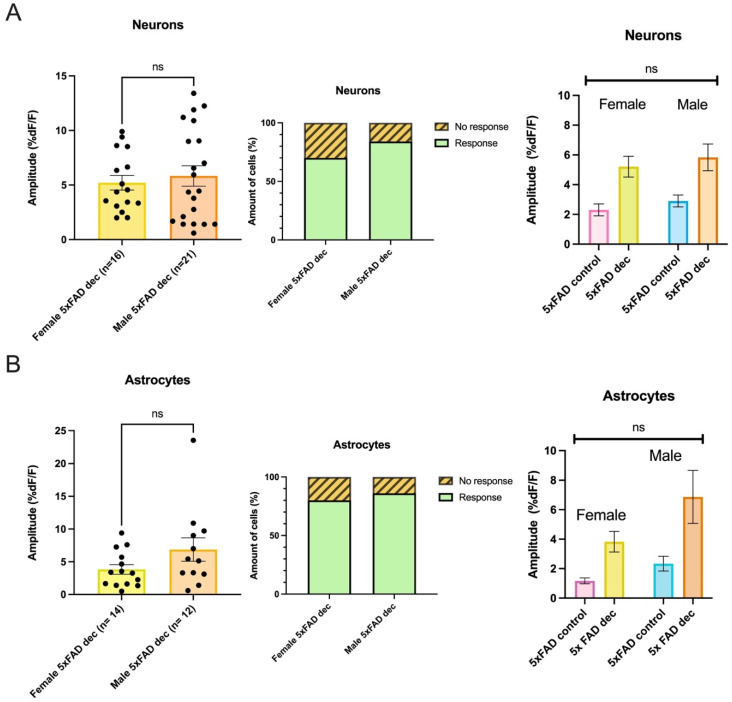
There was no difference between females and males in the amplitude of the response to AMPA in neurons or astrocytes following incubation in decanoic acid nor in the proportion responding. (**A**) The proportion of neurons responding and the amplitude of response to AMPA in slices incubated in decanoic acid did not differ between the female and male 5xFAD genotype. Further, there was no difference between females and males in the relative fold change in amplitude from control conditions in the 5xFAD neurons when decanoic acid was present (Two-way ANOVA, neurons: *p* = 0.9793). (**B**) The response proportion and the amplitude of the response to AMPA in presence of decanoic acid also did not differ in astrocytes between females and males, and there was no difference in the relative fold change in amplitude in presence of decanoic acid when compared to control conditions in the 5xFAD (Two-way ANOVA, Astrocytes: *p* = 0.3534). ns indicates no significance.

As expected based on their relative similarity, when we compared the amplitude of the AMPA-induced calcium change in slices preincubated in decanoic acid, we did not see a significant difference in the amplitude of the rise based on sex in neurons (average amplitudes: Female 5xFAD_Dec_: 5.21 ± 0.7 %DF/F, n = 16, Male 5xFAD_Dec_: 5.84 ± 0.9 %DF/F, n = 21; Mann–Whitney test: *p* > 0.9999; Figure 6A). Similarly, there was not a significant difference observed when rises elicited in female and male astrocytes in slices preincubated in decanoic acid were compared (average amplitudes: Female 5xFAD_Dec_: 3.83 ± 0.7 %DF/F, n = 14, Male 5xFAD_Dec_: 6.87 ± 1.8 %DF/F, n = 12; Mann–Whitney test: *p* = 0.1976; Figure 6B). There was no significant interaction between sex and treatment on the amplitude of AMPA-induces rises in neurons or astrocytes, suggesting that the degree by which decanoic acid enhanced AMPA-mediated transients was similar in 5xFAD females and males (Two-way ANOVA, neurons: *p* = 0.9793; astrocytes: *p* = 0.3534; Figure 6A,B). The proportions of responding neurons (Female 5xFAD_Dec_: Response n = 26, No response n = 11, Male 5xFAD_Dec_: Response n = 25, No response n = 5, Fisher’s exact test, *p* = 0.2584; Figure 6A), and astrocytes (Female 5xFAD_Dec_: Response n = 16, No response n = 4, Male 5xFAD_Dec_: Response n = 26, No response n = 5, Fisher’s exact test, *p* = 0.7241; Figure 6B) in the 5xFAD animal did not differ in decanoic acid conditions when compared to proportions of responses seen in absence of incubation.

### 3.8. Decanoic Acid Pre-Treatment Does Not Result in Significantly Heightened Calcium Responses in WT Astrocytes and Neurons in Male and Females

**Figure 7:**
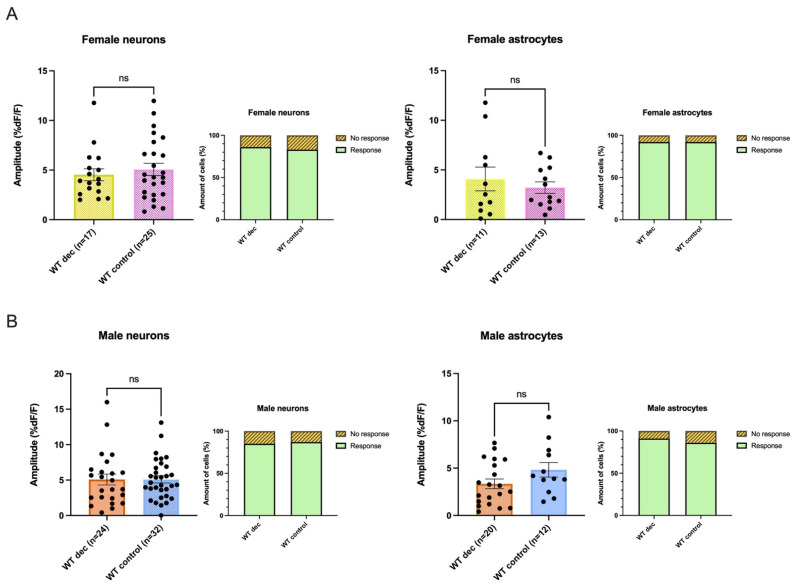
The effect of decanoic acid in altering AMPA-mediated calcium rises was not seen in neurons or astrocytes in either females or male wildtype (WT). (**A**) Neither the proportion responding nor the amplitude of response to AMPA differed in WT female neurons between responses seen in conditions when decanoic acid was present when compared to responses seen in control conditions. (**B**) Similarly, there was no effect of decanoic acid on the proportion responding or the amplitude of the response to AMPA in the male WT when compared to control conditions. ns indicates no significance.

In contrast to what had been seen in cells from 5xFAD slices, incubation in decanoic acid did not result in a significant change in either the proportion responding or the amplitude of AMPA-mediated rises in neurons or astrocytes from WT slices in either males or females. In female WT, the average amplitude of the calcium response in slices in which preincubation of decanoic acid was present was 4.53 ± 0.6 %DF/F (n = 17), which did not differ from the average amplitude of 5.066 ± 0.6 %DF/F, (n = 25), which had been seen in conditions absent of decanoic acid (Mann-Whitney test: *p* = 0.6300) (Figure 7A). In the female WT, astrocytes from slices preincubated in decanoic acid did not show a different amplitude from that seen in slices that were not exposed to decanoic acid (average amplitudes: WT_Dec_: 4.085 ± 1.2 %DF/F, n= 11, WT_Control_: 3.22 ± 0.6 %DF/F, n = 13; Unpaired Student’s *t*-test: *p* = 0.4994; Figure 7A). Furthermore, the proportion of responsive neurons (WT_Dec_: Response n = 24, No response n = 4, WT_Control_: Response n = 25, No response n = 5, Fisher’s exact test: *p* = 1.000) and astrocytes (WT_Dec_: Response n = 11, No response n = 1, WT_Control_: Response n = 13, No response n = 1, Fisher’s exact test: *p* = 1.000) in female WT exposed to decanoic acid did not differ from those in female WT mice that were not similarly exposed (Figure 7A).

In male WT neurons, the average amplitude of the AMPA-induced transient seen following preincubation in decanoic acid was 5.08 ± 0.8 %DF/F (n = 24), which did not differ from that seen in control conditions in the WT (5.10 ± 0.5 %DF/F, n = 32; Mann–Whitney test: *p* = 0.6514; Figure 7B). Further, no significant difference was observed in decanoic acid exposure in calcium responses elicited by AMPA in CA1 astrocytes in male WT (average amplitudes: WT_Dec_: 3.34 ± 0.5 %DF/F, n = 20, WT_Control_: 4.83 ± 0.8 %DF/F, n = 12; Unpaired Student’s *t*-test: *p* = 0.1059; Figure 7B). In addition, there was no difference between the proportion of cells in the male hippocampus responding to AMPA under decanoic acid preincubation conditions compared to those responding without this exposure (neurons: WT_Dec_: Response n = 33, No response n = 6. WT_Control_: Response n = 34, No response n = 5, Fisher’s exact test: *p* = 1.000; astrocytes: WT_Dec_: Response n = 21, No response n = 2, WT_Control_: Response n = 12, No response n = 2, Fisher’s exact test: *p* = 0.6246; Figure 7B).

The proportion of WT female cells responding to AMPA also did not significantly vary when decanoic acid was present when compared to the proportion of WT neurons responding in presence of decanoic acid in males (Female WT _Dec_: Response n = 24, No response n = 4, Male WT _Dec_: Response n = 33, No response n = 6, Fisher’s exact test, *p* = 1.000) or astrocytes (Female WT _Dec_: Response n = 11, no response n = 1, Male WT _Dec_: Response n = 21, No response n = 2, Fisher’s exact test, *p* = 1.000).

### 3.9. There Was No Significant Difference between 5xFAD AMPA Responses following Decanoic Acid Exposure When Compared to WT in Females or in Males, Suggesting That Decanoic Acid Rescues Altered Calcium Responses in 5xFAD Neurons and Astrocytes in Both Females and Males

In our last analysis, while we had seen that decanoic acid had a significant effect in increasing calcium transients in a genotype-specific manner, and this enhancement occurred in both 5xFAD females and males, we wished to determine whether rescue of the lower calcium transients seen in 5xFAD animals had been induced by the treatment; that is, could decanoic acid restore calcium levels to those seen in the WT? Accordingly, we compared the response in the 5xFAD neurons and astrocytes following preincubation with decanoic acid with that seen in WT cells and 5xFAD control cells. In females, a significant difference was detected when amplitudes from the three groups of neurons and the three groups of astrocytes were compared, and a significant difference was also seen in males when the three treatment groups were compared in neurons and in astrocytes (Table 1).

**Figure 8:**
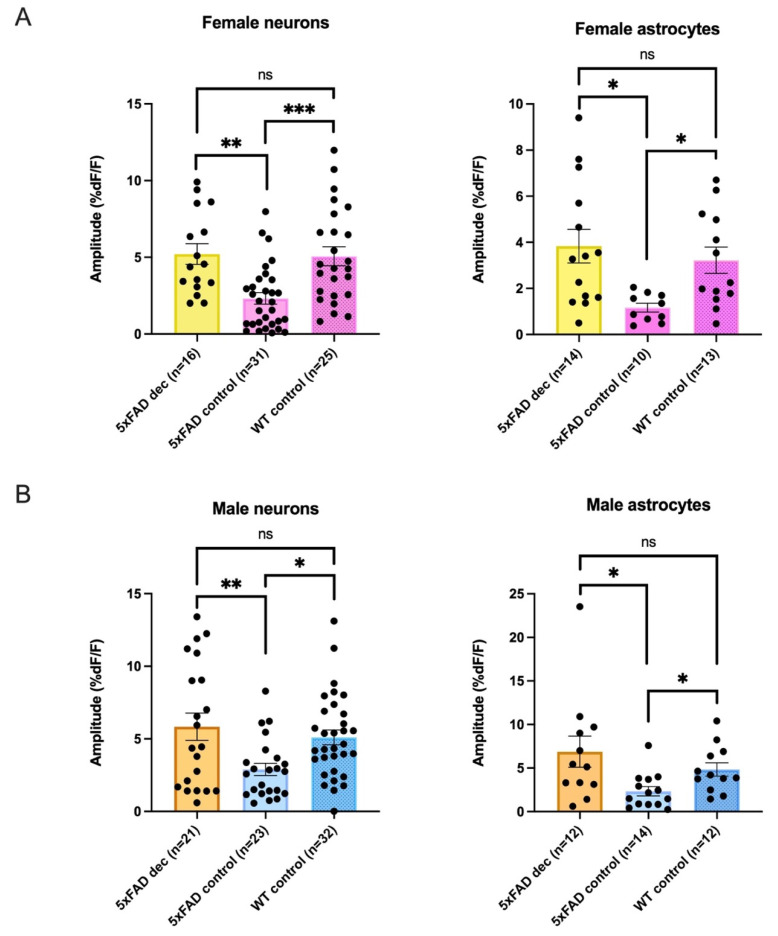
Decanoic acid rescues the reduction in AMPA-mediated calcium seen in neurons and astrocytes of the 5xFAD genotype and restores the amplitude to that seen in the wildtype (WT) in both sexes. (**A**) There was no difference between the amplitude of AMPA-mediated calcium transients in neurons and astrocytes of the female 5xFAD genotype when incubated in decanoic acid when compared to transients seen in the WT. (**B**) There was also no difference in the amplitude of the AMPA-mediated calcium transients elicited in neurons and astrocytes of slices from the male 5xFAD animal that were incubated in decanoic acid when compared to transients seen in the WT. The significance level is presented above the bars with asterisks denoting * *p* < 0.05, ** *p* < 0.01, and *** *p* < 0.001 with ns indicating no significance.

Because we noted a significance difference in neurons and astrocytes in both females and males when comparing the 5xFAD mouse with and without decanoic acid and the wildtype, we then conducted post hoc testing to see between which groups a difference was present. Remarkably, in post hoc testing we found no significant difference in neurons from females or males in AMPA-transients when the amplitudes were compared between 5xFAD neurons that had been preincubated in decanoic acid and WT (Female neurons: 5xFAD_Dec_: n = 16, WT control: n = 25, post hoc Dunn’s test *p* ≥ 0.9999; Male neurons: 5xFAD_Dec_: n = 21, WT_Control_: n = 32, post hoc Dunn’s test, *p* ≥ 0.9999; Figure 8A,B). As expected, there was a difference in both females and males between the 5xFAD neurons exposed to decanoic acid and the 5xFAD neurons that were not exposed to the MCFA (Female neurons: 5xFAD_Dec_: n = 16, 5xFAD_Control_: n = 31, *p* = 0.0015; Male neurons: 5xFAD_Dec_: n = 21, 5xFAD_Control_: n = 23; *p* = 0.0290; Figure 8). Also expected was the difference noted in the AMPA-mediated calcium amplitude in both females and males between the 5xFAD neurons not exposed to decanoic acid and the WT (Female neurons: 5xFAD_Control_: n = 31, WT_Control_: n = 25, *p* = 0.0008; Male neurons: 5xFAD_Control_: n = 23, WT_Control_: n = 32, *p* = 0.0107; Figure 8A,B).

Similarly, when astrocytes were compared across the three groups in post hoc testing, a difference was detected in both females and males between the 5xFAD control group and the WT (Female astrocytes: 5xFAD_Control_: n = 10, WT_Control_: n = 13, post hoc Dunn’s test *p* = 0.0272; Male astrocytes: 5xFAD_Control_: n = 14, WT_Control_: n = 12, post hoc Dunn’s test, *p* = 0.0468; Figure 8), as well as between the 5xFAD groups with and without preincubation in decanoic acid (Female astrocytes: 5xFAD_Dec_: n = 14, 5xFAD_Control_: n = 10, post hoc Dunn’s test, *p* = 0.0100; Male astrocytes: 5xFAD_Dec_: n = 12, 5xFAD_Control_: n = 14, post hoc Dunn’s test, *p* = 0.0206; Figure 8A,B). Importantly, there was no difference between the 5xFAD group exposed to decanoic acid and the WT (Female astrocytes: 5xFAD_Dec_: n = 14, WT_Control_: n = 13, post hoc Dunn’s test, *p* ≥ 0.9999; Male astrocytes: 5xFAD_Dec_: n = 12, WT_Control_: n = 12, post hoc Dunn’s test, *p* ≥ 0.9999; Figure 8A,B).

When taken together, our data suggest that preincubation with decanoic acid alters AMPA-mediated calcium rises and restores them to levels that do not differ from those seen in the WT, which raises the intriguing speculation that decanoic acid supplementation might have a therapeutic effect on neurons and astrocytes in the hippocampus specifically in the scenario of altered Aβ levels.

## 4. Discussion

### 4.1. Summary of Data

In this study, we show that AMPA-mediated calcium levels in hippocampal neurons and astrocytes of 5xFAD mice were 36% and 47.6%, respectively, than those seen in control WT animals at one month of age. Smaller rises in calcium in the 5xFAD animal did not depend on sex, as shown in a cohort that was 8–10 weeks of age, as reductions seen in the 5xFAD animal were similar in neurons and astrocytes in both females and males. AMPA-mediated transients in 5xFAD or WT CA1 neurons and astrocytes did not differ between females and males, which was unexpected. Excitingly, we found that preincubation in decanoic acid restored the amplitude of calcium transients back to levels seen in controls in both female and male CA1 neurons and astrocytes.

### 4.2. AMPA Mediated Transients Are Lower in Both Female and Male 5xFAD

AMPA-mediated calcium was lower in 5xFAD animals compared to WT controls, and when females and males were analyzed separately, there was no qualitative difference in this genotype difference. Accordingly, our data suggest that the presence of soluble Aβ is associated with alterations in AMPA-mediated calcium that are not sex-based. However, our data do not elucidate what molecular mechanisms might be altered by the presence of Aβ. AMPA-mediated calcium likely sources from multiple effectors [35,36]. Any of the effectors, which are activated subsequent to agonism of the AMPA receptor, could be differentially affected by Aβ. As AMPA receptors are permeable to calcium depending on the subunit composition, Aβ could lead to reductions in calcium entering via AMPA receptors if it is altering the subunit composition or number of AMPA receptors. As the disease progresses, a higher amount of accumulated Aβ occurs in the brain, leading to AMPA receptors being constantly endocytosed and removed from the postsynaptic membrane [37]. While it is only an indirect measure, our data showing no change in proportions of cells responding to AMPA suggests that there is not a great loss of total AMPA receptors upon high Aβ load, at least in the two-month-old 5xFAD female or male hippocampus. However, our data are consistent with changes in subunit composition. AMPA receptors are tetrameric and comprised of an obligatory GluA1 subunit, most often in combination with a GluA2 subunit. AMPA receptors, which lack GluA2 receptors, are highly calcium permeable. Our data could be explained by an alteration in the presence of GluA2 in the 5xFAD hippocampus, which is consistent with findings that Aβ alters AMPA receptor subunit composition. When Aβ is bound to GluA2, an internalization and degradation of the receptor occurs. Aβ induces aggregation of GluA2 while also increasing the loss of surface GluA1 subunits in AMPA receptors, leading to an overall decrease of Ca^2+^ influx into neurons [38]. Further, oligomeric Aβ reduces the density of postsynaptic AMPA receptors through endocytosis of GluA1 expressed on the surface of dendritic spines [39,40,41,42,43,44], and this downregulation was associated with a partial reduction in AMPA receptor-mediated synaptic transmission in CA1 pyramidal neurons presumably accompanied by a more modest calcium influx [45]. However, likely in a compensatory mechanism, in familial forms of AD, GluA2 is trafficked to the cell surface, resulting in the restoration of AMPA receptor density, presumably with a lowering of overall calcium permeability [45]. When taken together, our data are consistent with compensatory increases in the presence of GluA2 subunit in AMPA receptors in the 5xFAD hippocampus, resulting in smaller calcium rises upon receptor activation by AMPA. However, as our conclusion is speculative, protein studies should be conducted to determine whether subunits are changing in the composition of the AMPA receptors in CA1 of the 5xFAD animal or whether total numbers of these receptors are reduced.

Another explanation for lower calcium responses in the 5xFAD animal is that altered Aβ could induce changes in downstream effectors that are activated following AMPA stimulation. AMPA receptors mediate depolarization of the membrane, which can lead to indirect rises in intracellular calcium through activation of voltage-operated calcium channels (VOCCs) or activation of NMDA receptors due to the removal of a magnesium block in the pore. We do not believe that the magnitude of the depolarization following AMPA receptor activation is greatly altered in two-month-old 5xFAD animals. In an earlier study using patch clamp electrophysiology, we showed that the amplitude and frequency of fast EPSCs putatively mediated by glutamate acting at AMPA receptors were significantly greater in 5xFAD CA1 neurons in males [34]. These findings are not consistent with the explanation that lower calcium rises seen in the present study are due to a lower depolarization mediated by the AMPA receptor, leading to reduced activation of VOCC and NMDA receptors. However, Aβ has been found to decrease the amplitude of NMDA-mediated currents, which includes a reduction in calcium influx via the NMDA receptor [46,47]. Effects on NMDA currents and calcium in Aβ-treated cells were associated with a decrease in the number of postsynaptic NMDA receptors at glutamatergic synapses [47], suggesting that lower rises seen in our study could be due to changes in NMDA receptors. Accordingly, future studies will need to examine whether Aβ has actions on mediators activated down stream of AMPA receptor stimulation, which could include changes in NMDA receptor functionality that could lead to the lower rises in calcium seen in our study.

### 4.3. Decanoic Acid Restores the Amplitude of AMPA-Mediated Transients to WT Levels

While we could not identify the molecular mechanism by which lower calcium transients were elicited by AMPA in cells of the 5xFAD hippocampus, we were able to show that decanoic acid could restore AMPA transients in both neurons and astrocytes to levels seen in control animals. We have recently shown that decanoic acid is metabolized in astrocytes, where it serves glutamine synthesis [27]. Glutamine may, in turn, support neuronal amino acid and energy metabolism [48]. The ability of decanoic acid to support glutamine synthesis, as well as glial and neuronal energy metabolism, may be particularly favorable in pathological conditions where cellular bioenergetics are impaired, such as the Aβ-burdened brain. In line with this, we have previously shown that astrocyte and neuronal metabolism is hindered in the 5xFAD brain [34]. Thus, it can be speculated that the effect of decanoic acid, at least the metabolic effect supporting astrocyte and neuronal metabolism, may be selectively noticeable when brain metabolism is impaired. In support of this, we found that decanoic acid selectively heightened calcium responses in 5xFAD astrocytes and neurons, whereas calcium responses were not altered in matched WT counterparts.

While decanoic acid could exert metabolic actions, it also could function as a signaling molecule. Many protein targets of decanoic acid, when activated, could alter mechanisms involved in calcium signaling in neurons. For example, MCFAs can stimulate the GPR84, the mammalian target of the rapamycin (mTOR) pathway, as well as the Free Fatty Acid type I receptor (FFA1R), among others. Interestingly, MCFAs induce rises in calcium in pancreatic β-cells by acting as stimulators of FFAR1, which leads to activation of the G_q_ family of G-proteins [49]. FFA1Rs have been primarily studied for their actions in the periphery; however, they are also found in the CNS, including neurons and astrocytes of the hippocampal dentate gyrus [50], suggesting that free fatty acids could alter hippocampal cellular functioning. While TUG-770, a potent FFAR1 agonist, was capable of leading to rises in calcium and stimulating insulin secretion from pancreatic β-cells [49], this point must be explicitly examined in hippocampal CA1 neurons as effects could be cell type-specific.

Decanoic acid has been shown to inhibit the mechanistic mTOR complex 1 (mTORC1) [51]. As mTOR activation regulates AMPA receptors by increasing synaptic expression of GluA1/GluA2 [52], inhibition of this pathway by decanoic acid could result in higher expression of GluA2-lacking AMPA receptors reflected as higher amplitudes of calcium responses. Notably, studies have found mTOR to be hyperactive in both in vitro and in vivo AD models, partly by Aβ, since a reduction of Aβ in the 3xTg-AD mouse model showed a reverse in mTOR hyperactivity [53].

Decanoic acid acts as an agonist at the GRP84 receptor, and the agonism of GRP84 has been shown to lead to alterations in the morphology and motility of microglia [54], which would be expected to influence astrocytic function [55]. As both microglia and astrocytes control the function of PSD-95, a scaffolding protein underlying anchoring of both AMPA and NMDA receptors, and PSD95 has been shown to protect NMDA and AMPA receptors on the synapses from Aβ toxicity [56], agonism of GRP84 by decanoic acid perhaps could bolster the functionality of PSD-95, leading to larger rises in calcium-mediated by glutamatergic receptors. Future studies should explore the mechanisms by which decanoic acid could lead to increases in AMPA-mediated transients in neurons and astrocytes in the hippocampus. While effects could be metabolic, as protective effects of MCFAs have poorly correlated with ketone levels [13], while speculative, it is possible that the decanoic acid effects seen in our study are due to actions on signaling. Future studies should include characterization of effects of decanoic acid on signaling pathways in hippocampal neurons and astrocytes, which have been shown to be affected by decanoic acid in other cell types (i.e., FFA1R, GRP84, mTOR).

It is worth noting that decanoic acid has been shown in oocytes in which AMPA receptors have been expressed to act in a voltage- and subunit-dependent manner as a non-competitive antagonist of the AMPA receptor, with a binding site likely at the GluA2 subunit [57]. Further, a circa 15 min application of 300 µM decanoic acid reduced seizure activity in a rat hippocampal slice model of epileptic activity with strong actions on inhibition of excitatory, but not inhibitory, neurotransmission [57]. Decanoic acid-mediated antagonism of the AMPA receptor is not consistent with our data on the restoration of AMPA-mediated calcium rises in the 5xFAD animal, and in the WT animal, we did not see any evidence of AMPA receptor antagonism. However, while our concentration is similar to that used in [57], our data are not directly comparable to those in that study as we utilized one-hour incubation times. While decanoic acid might have had a fast-acting antagonistic action, as shown in [57], it is feasible that the incubation times we used were sufficient to induce metabolic or signalling effects that could involve or invoke changes in proteins, leading to the enhancement of AMPA-mediated calcium. A non-mutually exclusive possibility is that glutamate hyperexcitability in the 5xFAD hippocampus seen in our previous work could be acutely affected by the inhibitory actions of decanoic acid on the AMPA receptor, which could contribute to therapeutic effects [58].

### 4.4. There Were No Sex-Differences in AMPA-Mediated Transients in the 5xFAD Mouse, and Dec Showed a Sex-Independent Effect on AMPA-Transients

In our study, we hypothesized that the presence of high levels of Aβ would lead to functional changes in hippocampal cells, which would be reflected as alterations in AMPA-mediated calcium rise, and we expected to see a sex difference in this change based on cellular and epidemiological studies. Epidemiological studies have suggested that females are affected more severely and frequently by AD when compared to men, suggesting greater actions of Aβ in the female brain [3,4,5], although this has been debated [59,60,61]. Female 5xFAD mice exhibit more severe amyloid accumulation than male mice, especially in the later stages of this mouse model of AD [8]. Further, females exhibit lower levels of GluA2-containing AMPA-receptor subunits, suggesting higher calcium permeability through AMPA receptors, which has been suggested to confer a higher vulnerability to excitotoxicity [62]. In our study, no sex-based difference in AMPA-mediated calcium responses was observed in neurons of female 5xFAD animals when compared to responses in male 5xFAD mice.

While we cannot explain why we saw no sex-based differences in AMPA-mediated rises, an unidentified compensatory mechanism could be more prevalent in females earlier in the disease, and it is possible that this compensatory response wanes with aging and progression of the disease pathology. This is consistent with studies that have found compensatory responses to occur early during neuronal distress, resulting in the strengthening of synaptic activity and activation of protective glial cells to compensate for initial damage and induce resilience to the pathology progression. A sex-based differential response to inflammation induced by a variety of inflammatory factors has been noted with astrocytes in females exhibiting a higher resistance to oxidant-induced cell-death (i.e., by ROS) than male astrocytes, and perhaps this could counteract the negative effects of Aβ on other processes which could have affected AMPA-mediated calcium [63]. Further, astrocytes in females have been shown to confer more neuroprotection against excitotoxicity due to an enhanced glutamate-uptake phenotype, which protects motor neurons from excessive synaptic glutamate [64]. A non-mutually exclusive possibility is that estrogen could play a special protective role in females. Estrogen has been shown to reduce Aβ production by the non-amyloidogenic pathway by reducing β-secretase levels and promoting Aβ clearance [65]. Regardless of the specific mechanisms that could lead to neuroprotection, neuronal activity can, over time, exacerbate neuronal damage due to an increased rate of neurotransmitter release, so these protective mechanisms are time-limited and are likely to play less of a role as the aging process proceeds [66]. In this context, when considering a lack of a sex difference, it is worth noting that 5xFAD mice used in the sex-based comparison study were 8-10 weeks of age, which could explain why no sex differences were seen. While female 5xFAD mice exhibit greater pathology than 5xFAD male mice and several markers for neuroinflammation (such as macrophage expressed 1 (Mpeg1)) are observed that were increased in both male and female 5xFAD and controls at 2 months of age, these markers were more abundant in female 5xFAD compared to male 5xFAD at 4 months of age [67]. This study suggests that old female 5xFAD mice might be more prone to neuroinflammation and exhibit greater AD pathology than old 5xFAD male mice, so differences in AMPA-mediated events might be seen at later stages. Lending support to this conclusion, female mice exhibit a higher γ-secretase activity in aged brain compared to male mice, indicating that aging can affect γ-secretase activity which would affect formation of Aβ peptides [68]. Nevertheless, while we did not see the expected sex-based difference in AMPA-mediated transients in 5xFAD animals, we found that decanoic acid had a restorative effect in both sexes in the early stage of Aβ accumulation which was qualitatively similar in that it restored amplitudes of calcium rises back to levels seen in WT animals. This suggests that decanoic acid should be explored for its actions in both sexes, despite potential differences in the epidemiology and progression of the disease.

### 4.5. Caveats

When using the 5xFAD AD mouse model, which expresses five different mutations (APP_K670N/M671L,I716V,V717I_, PS1_M146L,L286V_) [8], consideration must be made that no incidence of the occurrence of these five mutations simultaneously has been reported in human AD. Individually, the K670N/M671L substitution within PS1 either elevates the production of total Aβ or specifically Aβ_42_ [69], as well as the individual I716V and V717I mutations within APP elevate the production of specifically Aβ_42_ [70]. The combination of these mutations into one line additively doubles the level of specifically Aβ_42_ [70]. Mutations in this mouse model are from those seen in the familiar form of AD, which is a direct result of over-expression of Aβ, and do not reflect sporadic AD that is likely due to the failure of multiple factors to clear Aβ from the brain. As most AD cases are sporadic, findings obtained from this model could be relevant to only a minority of AD cases [71]. Further, the Aβ42/Aβ40 ratio is higher in the 5xFAD mouse model than in human AD brains, suggesting a higher Aβ42 toxicity in the mouse model [8], and this model only possesses Aβ and, therefore, lacks tau. When taken together, these disadvantages limit translatability to the human disease and raise a concern that findings might be less replicable in humans and treatments such as decanoic acid, which could have putative therapeutic actions in the mouse, might be less likely to show effects in humans. Regardless, the rapid amyloid pathology of the 5xFAD mouse model confers advantages to its use in AD research. While Aβ accumulates very slowly over a 15 year period in primates [72], the 5xFAD model reduces the span of time required for investigating mechanisms that are likely to involve Aβ. In addition, the 5xFAD model does exhibit many of the fundamental characteristics of AD, including gliosis, neuron loss, and interneuronal Aβ that many other transgenic mice of AD do not possess [8]. Therefore, this model presents an ideal biological system for the quick evaluation of novel hypotheses about Aβ-associated functional changes and for the rapid assessment of potential therapeutics [8].

The import of including females and males as separate populations in a cellular study of hippocampal function has gained wide recognition in the neuroscience community, which gives value to sex-separated studies. As the estrus cycle in females has a dramatic effect on varying the stoichiometry and numbers of AMPA receptors across the cycle [73,74], we were surprised to find a lack of differences in AMPA-mediated calcium rises when we compared them between WT females who were between 8–10 weeks of age and age-matched males. However, our data are difficult to interpret as an additional caveat of our study is that we had no control for the effect of the estrus cycle on AMPA-mediated responses. Many studies control for the effects of the estrus cycle by ovariectomizing their animals, which we did not do. Studies that have seen sex differences across the estrus cycle in hippocampal calcium-dependent processes, which could involve AMPA receptors, did not look at calcium, and thus, differences in experimental design could explain why we failed to see any differences [75]. Regardless, while our findings that there were no differences in AMPA-mediated transients based on sex should be taken with caution in light of the complexity of differences in the hippocampus in function [76], we did not see differences in the effects of decanoic acid in age- and sex-matched 5xFAD animals, which indicates that decanoic acid has actions in both females and males.

### 4.6. Conclusions and Significance

This study reports that in a mouse model of AD, young female and male mice showed reduced levels of rises in calcium in both CA1 neurons and astrocytes upon AMPA-receptor stimulation. As calcium is a vital signaling molecule, reductions in intracellular levels indicate a functional impact in the 5xFAD AD model, which would be expected to have profound effects on normal signaling, which could impact cognitive behaviors in which the hippocampus is involved. We found that decanoic acid restored the level of AMPA-mediated calcium levels in the CA1 area of the hippocampus in neurons and astrocytes in both sexes to those seen in age and sex-matched control animals. While the mechanisms by which decanoic acid restored AMPA-mediated calcium transients in this AD mouse model remain to be elucidated, when taken together, the data suggest that one future treatment strategy for enhancing hippocampal-involved cognitive behaviors involving dysfunctions of the AMPA receptor in AD could be by enhancement of diet with decanoic acid and that this intervention could be relevant to both females and males at least prior to significant progression of the disease.

## Figures and Tables

**Table 1:** Comparision of average amplitudes of rises in fluorescence (in %DF/F) in neurons and astrocytes in female and male 5xFAD animals treated with decanoic acid, 5xFAD control animals and wildtype controls.

Female Average Amplitudes %DF/F				
Neurons	5xFAD_Dec_	5xFAD_Control_:	WT_Control_	Kruskal-Wallis
	5.21 ± 0.7 %DF/F n = 16	2.32 ± 0.4 %DF/F n = 31	5.07 ± 0.6 %DF/F n = 25	*p* = 0.0001
Astrocytes *	3.83 ± 0.7 %DF/F n = 14	1.17 ± 0.2 %DF/F n = 10	3.22 ± 0.6 %DF/F n = 13	*p* = 0.0071
**Male Average Amplitudes %DF/F**				
**Neurons**	**5xFAD_Dec_**	**5xFAD_Control_:**	**WT_Control_**	**Kruskal-Wallis**
	5.84 ± 0.9 %DF/F n = 21	2.90 ± 0.4 %DF/F n = 23	5.10 ± 0.5 %DF/F n = 32	*p* = 0.0066
Astrocytes *	6.87 ± 1.8 %DF/F n = 12	2.34 ± 0.5 %DF/F n = 14	4.83 ± 0.8 %DF/F n = 12	*p* = 0.0108

* Indicates significance in Kruskal–Wallis test.

## Data Availability

All data are available upon reasonable request made to the corresponding author.
